# Antiviral effects of the petroleum ether extract of *Tournefortia sibirica L*. against enterovirus 71 infection *in vitro* and *in vivo*

**DOI:** 10.3389/fphar.2022.999798

**Published:** 2022-11-29

**Authors:** Xinyu Huang, Jiemin Li, Yan Hong, Chenghan Jiang, Jiaxin Wu, Min Wu, Rui Sheng, Hongtao Liu, Jie Sun, Ying Xin, Weiheng Su

**Affiliations:** ^1^ National Engineering Laboratory for AIDS Vaccine, School of Life Sciences, Jilin University, Changchun, China; ^2^ Key Laboratory for Mongolian Medicine R&D Engineering of the Ministry of Education, School of Mongolian Medicine and Pharmacy, Inner Mongolia Minzu University, Tongliao, China; ^3^ College of Agriculture, Yanbian University, Yanji, China; ^4^ Key Laboratory for Molecular Enzymology and Engineering of the Ministry of Education, School of Life Sciences, Jilin University, Changchun, China

**Keywords:** enterovirus 71 (EV71), hand, foot, and mouth disease, *Tournefortia sibirica L.*, antiviral herbs, petroleum ether extract

## Abstract

Enterovirus 71 (EV71) is the major cause of severe hand, foot, and mouth disease (HFMD). Compared to other HFMD pathogens, like coxsackievirus A16 (CVA16), EV71 can invade the central nervous system and cause permanent damage. At present, there are no available antivirals against EV71 for clinical treatment. Herein, multiple Chinese botanical drugs were collected, and 47 types of botanical extracts were extracted using aqueous solutions and organic solvents. Based on the cytopathic effect inhibition assay, petroleum ether extract of *Tournefortia sibirica L.* (PE-TS) demonstrated 97.25% and 94.75% inhibition rates for EV71 infection (at 250 μg/ml) and CVA16 infection (at 125 μg/ml), respectively, with low cytotoxicity. Preliminary mechanistic studies showed that PE-TS inhibits replication of EV71 genomic RNA and synthesis of the EV71 protein. The released extracellular EV71 progeny virus titer decreased by 3.75 lg under PE-TS treatment. Furthermore, using a newborn mouse model, PE-TS treatment protected 70% and 66.7% of mice from lethal dose EV71 intracranial challenge *via* administration of intraperitoneal injection at 0.4 mg/g and direct lavage at 0.8 mg/g, respectively. The chemical constituents of the PE-TS were analyzed by Gas Chromatography-Mass Spectrometer (GC-MS), and a total of 60 compounds were identified. Compound-target network analysis and molecular docking implied potential bioactive compounds and their protein targets against EV71 associated pathology. The present study identified antiviral effects of PE-TS against EV71/CVA16 infection *in vitro* and EV71 infection *in vivo*, providing a potential antiviral botanical drug extract candidate for HFMD drug development.

## Introduction

Hand, foot, and mouth disease (HFMD) is a global contagious disease which main infect infants and children. HFMD is clinically characterized by papules or a blister rash on the hands, feet, mouth, or other parts of the body (Wong et al., 2010). Severe HFMD cases may progress to aseptic meningitis, neuronal pulmonary edema, myocarditis, and even death (Secher et al., 2015). The major pathogens of HFMD are enterovirus A71 (EV71), coxsackievirus A16 (CVA16), and so on (Saguil et al., 2019). These enteroviruses are non-enveloped viruses with a single positive-strand RNA genome. Severe and fatal HFMD cases are always caused by EV71 infection (Ji et al., 2019), which has long been considered a major target for vaccine development and antiviral treatment. The CVA16 incidence rates are approximately 25% in China, and its infection always causes mild symptoms (Ji et al., 2019).

Currently, vaccines remain the most effective means of preventing and controlling HFMD. Several inactivated EV71 vaccines have been marketed in China (He et al., 2020); however, there is no vaccine for protection against HFMD in the rest of the world. More importantly, there is no specific antiviral compound for the clinical treatment of HFMD. Ribavirin has been used clinically to alleviate the symptoms of HFMD caused by EV71, but its effectiveness is limited to the early stages of viral infection, and its side effects are relatively adverse to the prognosis and recovery of patients (Zhang et al., 2014). Rupintrivir and pleconaril have been used to treat patients at the early stages of HFMD in clinical trials, they have been effective in alleviating the symptoms but have induced significant side effects (Pourianfar and Grollo, 2015). We have identified the effective antiviral activities of luteolin, apigenin, kaempferol, formononetin, penduletin, Retro-2cycl, and Retro-2.1 against EV71 infection *in vitro* and in animal experiments (Chen et al., 2014; Xu et al., 2014; Dai et al., 2017; Dai et al., 2019). However, these compounds still have a long way to go towards clinical trials. Therefore, the development of effective drugs against EV71 is urgently required.

Traditional Chinese medicine mainly consists of botanical drugs (root, stem, leaf and fruit) and mineral drugs. Chinese botanical drugs have shown potential for antiviral treatment because of their safety, reliability, and lack of adverse side effects. In clinical studies, the therapeutic effect of some traditional Chinese medicines on EV71 infection has been verified, such as Qingkailing, Xiyanping, and Lanchen Oral Solution (Shen et al., 2014; Li et al., 2021a). Administration of ribavirin and Xiyanping supplemented with retained enemas showed antipyretic and anti-eruption effects in children with HFMD (Yang and Huang, 2016). The administration Shuanghuanglian supplemented with retention enema also benefited the treatment of infantile HFMD (Zhang, 2009). Accordingly, identification and development of anti-EV71 medicines from herbal sources could be a time-saving and practical strategy.

Some herbs grown in Inner Mongolia of China, such as *Artemisia stechmanniana* Bess, *A. mongolica* (Fisch. ex Bess.) Nakai, *T. sibirica* , *Scutellaria scordifolia* Fisch. ex Schrank, *Artemisia ordosica* Krasch., *Artemisia austriaca* Jacq., *Aconitum septentrionale* Koelle, have been recorded for usage as anti-infection and anti-inflammation purpose in ancestral Mongolian medical books and still used currently in Mongolian community of China. These botanical drugs could be sources for discovery of novel antiviral constituents and even compounds; however, they have not been researched extensively. In this study, we screened 47 botanic extracts prepared from multiple botanical drugs collected from Inner Mongolia of China for antiviral activity against EV71 infection and identified the antiviral effects of petroleum ether extract of *T. sibirica*. (PE-TS) against EV71 *in vitro* and *in vivo*.

*T. sibirica*, which belongs to the family Boraginaceae, is a perennial botanical drug with a height of up to 30 cm and a slender rhizome. There are two species and one variety in China, which are distributed in the north and southeast regions, respectively. The entire *T. sibirica* plant is utilized in Inner Mongolia as a remedy for scrofula, eczema, sores, and ulcers (Wu, 2014; Ao and Na, 2015). To date, no study has shown that any part of *T. sibirica* produces inhibitory effects on enterovirus. The present study provides a novel natural product candidate for anti-EV71 drug development.

## Materials and methods

### Cells, viruses, and animals

Human malignant embryonic rhabdomyoma (RD) cells, susceptible to EV71, were maintained in our laboratory (Li et al., 2013). Cells were cultured in Dulbecco’s modified Eagle’s medium (DMEM, Invitrogen, United States) supplemented with 10% fetal bovine serum (FBS, Invitrogen) (DMEM-10% FBS) at 37°C with 5% CO_2_. The EV71 (GenBank accession No. KJ508817) was provided by the Chinese Center for Disease Control and Prevention. CVA16 (GenBank accession No. JF695003.1) was provided by the Henan Provincial Center for Disease Control and Prevention. The EV71-luciferase pseudovirus (EV71-luciferase) encoding a luciferase reporter gene in the viral genome was pre-packaged before the experiment, as previously described (Xu et al., 2014).

Female breeder specific-pathogen-free (SPF) grade BALB/c mice were purchased from the Liaoning Institute of Biological Products (China). Newborn BALB/c mice born within 24 h from breeder mice were selected as subjects for *in vivo* experiments. All experiments were performed in a BSL-2 facility with all experimental methods carried out in accordance with the regulations and guidelines set forth by the Animal Experiments Committee of Jilin University. The protocols for mouse experiments were approved by the Laboratory Animal Ethics Committee of the School of Life Science, Jilin University (Approval Number: 2018-nsfc010).

### Botanical drugs collection, identification and ethanolic extraction

All botanical drugs in this study were collected in the Korqin area of Tongliao City, Inner Mongolia, China, in July 2018, and were identified by Prof. Buhebateer working at Inner Mongolia Minzu University. A voucher (no. 20180720) was deposited at the School of Mongolian Medicine and Pharmacy, Inner Mongolia Minzu University. Luteolin purchased from Meilun Biotech Co., Ltd. (China) was used as a positive drug control *in vitro* (Xu et al., 2014).

Water and alcohol extracts of botanical drugs were prepared using water or ethanol to heat and reflux and then recover the solvent. Essential oils from botanical drugs were prepared by steam distillation. The dried ethanol extract was suspended in water and extracted sequentially using petroleum ether, dichloromethane, ethyl acetate, and n-butanol. The extracts of the botanical drugs were obtained after the solvent was recovered (ethanol, petroleum ether, dichloromethane, ethyl acetate, n-butanol; all were 100% pure and purchased from Beijing Chemical Works) at room temperature. All extracts were dissolved in DMSO to 2 mg/ml stocks and diluted with DMEM-2% FBS to working concentrations. To obtain the extract of *T. sibirica*, also named *Messerschmidia sibirica L.*, 2.0 kg whole plant was ground-dried, then. Was extracted with 95% ethanol (20 L) three times, for 4 h each time. Evaporation of the solvent under reduced pressure yielded the ethanol extract. The ethanol extracts (250.0 g) of *T. sibirica*. were mixed with water (mass–volume ratio of 1:2, g/mL), and petroleum ether was added each time (mass–volume ratio of 1:2, g/mL). The preferred number of extractions was between 25 and 30. All petroleum ether extracts were combined and recovered under reduced pressure to obtain a petroleum ether extract of *T. sibirica* (PE-TS) at a total amount of 47.2 g. The PE-TS was stored at −20°C until its use. The PE-TS was also dissolved in DMSO and DMEM-2% FBS, as described above.

### Cytopathic effect inhibition and cytotoxicity assays

Briefly, RD cells (5  × 10^3^ cells/well) were seeded in 96-well plates at 80% confluence. To determine the cytopathic effect (CPE) inhibition activity, the cells were treated with botanical drug extracts in DMEM-2% FBS solution at working concentrations of serial 2-fold dilutions (8,000–1.95 μg/ml). Meanwhile, the cells were infected with EV71 (MOI = 0.05) or CVA16 (MOI = 0.005) for 48 h at 37°C with 5% CO_2_. To determine the cytotoxicity, the cells were treated with only the botanical drug extracts in DMEM-2% FBS at serially diluted concentrations for 48 h. Cell viability was measured using a cell counting kit (CCK)-8 (Boster Biological Technology Co. Ltd., United States), and the OD490 was determined using a microplate reader (CliniBio 128C, BIOMAD). Antiviral activity was measured using the following equation: (T - Vc)/(Cc - Vc), where T, Vc, and Cc are the absorbance values of botanical drug extract-treated cells, virus control, and cell control, respectively. The 50% cytotoxicity concentration (CC_50_) and the 50% effective concentration (EC_50_) were analyzed using GraphPad Prism 5.0, as reported and calculated using regression analysis (Rudi et al., 1988). Selective index value was calculated by the ratio of CC_50_/EC_50_.

### Quantitative reverse transcription-polymerase chain reaction

RD cells at 90% confluence in 24-well plates were infected with EV71 (MOI = 0.2) in the presence of the PE-TS at a concentration of 250 μg/ml. Luteolin treatment (8.59 μg/ml) was used as a positive control. The supernatant was collected at 16 hpi for viral RNA extraction. EV71 RNA was extracted using the EasyPure^®^ Viral DNA/RNA Kit for Virus Detection (TRANSGEN Biotech Co., Ltd., Beijing, China). qRT-PCR was performed using the One Step SYBR^®^ PrimeScript™ RT-PCR Kit II (Takara Bio, Otsu, Japan) and the 5′ untranslated region (UTR) specific primers (sense 5′-TCC​TCC​GGC​CCC​TGA-3′ and antisense 5′-AAT​TGT​CAC​CAT​AAG​CAG​CCA-3′) as previously reported (Nijhuis et al., 2002) using the Bio-Rad CFX96 system (United States). Relative EV71 genomic RNA copies were normalized to the endogenous control (β-actin, sense 5′- CCA​CCA​TGT​ACC​CAG​GCA​TT-3′ and antisense 5′- CGG​ACT​CAT​CGT​ACT​CCT​GC-3′) using the 2^−ΔΔCt^ method (Livak and Schmittgen, 2001; Su et al., 2010).

### EV71-luciferase-based protein synthesis assay

RD cells (5 × 10^3^ cells/well) were seeded in 96-well plates, cultured to 90% confluence, treated with 250 μg/mL PE-TS or 8.59 μg/ml luteolin (positive control) in DMEM-2% FBS, and infected with EV71-luciferase (MOI = 0.5). Each concentration was set with three parallel wells and incubated at 37°C with 5% CO_2_. After infection for 1 h, the inoculum was replaced with the drugs at corresponding concentrations in DMEM-2% FBS. The supernatant was discarded at 16 hpi, and 100 μl of Bright-Glo™ luciferase reagent (Promega, United States) was added to each well. Luciferase activity was determined with the VICTOR X2 Multilabel Plate Reader (PerkinElmer, United States).

### Progeny virus yield assay

RD cells (2 × 10^4^ cells/well) were seeded in 24-well plates, cultured to 90% confluency, infected with EV71-WT (MOI = 1) and treated with 250 μg/mL PE-TS. After infection for 1 h, the inoculum was replaced with the drugs at corresponding concentrations in DMEM-2% FBS. At 16 hpi, cell supernatants were collected. Thereafter, the virus titers in the cell supernatants were determined using endpoint dilution assays, and the 50% tissue culture infectious dose (TCID_50_) was calculated using the Reed and Muench method (Lu et al., 2011).

### Antiviral efficacy of PE-TS against EV71 challenge in newborn mice

Newborn BALB/c mice (born within 24 h) (*n* = 9–11 per group) were intracerebrally challenged with EV71 at a lethal dose of 1.5 × 10^6^ TCID_50_ per mouse. Since the botanical extract PE-TS contained sticky insoluble matter after dissolution, PE-TS in the DMSO-water solution was filtered to exclude the influence of insoluble matter in the animal. The administration groups were set as follow: intraperitoneal injection of 0.4 mg/g or 0.13 mg/g filtered PE-TS, direct lavage of 0.4 mg/g or 0.13 mg/g filtered PE-TS, direct lavage of 0.8 mg/g or 0.26 mg/g unfiltered PE-TS. All extracts were added to sterile PBS supplemented with 10% DMSO, which was administrated once daily for seven consecutive days. Two groups were inoculated with sterile PBS supplemented with 10% DMSO as the uninfected control. The mice were monitored daily for 16 days to observe survival rate, clinical score, and body weight. Survival rate was estimated using the Kaplan-Meier method. Disease signs were evaluated to obtain a graded clinical score (0, healthy; 1, slow movement; 2, weakness in hind limbs; 3, paralysis in a single limb; 4, paralysis in two limbs; and 5, death) as described previously (Zhang et al., 2013).

### Gas chromatography-mass spectrometer analysis

GC-MS analysis of PE-TS was performed using GC-MS (Model: GC MS-5977B, Agilent, United States) equipped with a 325/350C HP-5 MS fused silica capillary column of 30 m length, 0.25 mm diameter and 0.25 µm film thickness. For GC-MS analysis, electron ionization system with ionization energy of 70 eV was used. The carrier gas used was helium (99.9%), at a constant flow rate of 1.0 ml/min. Injector and mass transfer line temperature were set to 270°C and 260°C, respectively. The oven temperature was set from 70°C to 325°C at 15°C/min for 5 min and finally raised to 325°C for 10 min. One microliter of the sample was injected in a split mode with a scan range of 50–1,000 m/z. The total running time of GC-MS was 33 min. The GC-MS total ion chromatograms of the chemical constituents of PE-TS were obtained, and each chromatographic peak was searched using the NIST17. L mass spectral library. The chemical components with a matching degree higher than 90% were selected according to the mass spectral data and relative retention time.

### Prediction of compound targets *via* network pharmacology

The volatile organic compounds of the PE-TS were harvested using the Traditional Chinese Medicine Systems Pharmacology Database and Analysis Platform (TCMSP, https://tcmspw.com/tcmsp.php). The compounds with oral bioavailability ≥30% and drug-likeness ≥ 0.18 were considered as potential active compounds. Targets of the active compounds of volatile organic compounds of the PE-TS were selected through both the similarity ensemble approach (SEA) (http://sea.bkslab.org/) and Swiss target prediction (STP) (http://www.swisstargetprediction.ch/) in “*Homo Sapiens*” mode. HFMD-related *H. sapiens* targets were searched in the GeneCards platform (https://www.genecards.org/) and DisGeNET (https://www.disgenet.org/search). Then, these collected targets were merged, and duplicates were removed. The overlapping targets between potential active compounds of the PE-TS and HFMD targets were identified. The network diagram between compounds and targets was established using Cytoscape 3.7.2 (http://www.cytoscape.org/). The overlapping targets were input into String 11.0 (https://string-db.org/) to obtain the interaction results among the targets, and the visual analysis results were obtained by Cytoscape 3.7.2.

### Molecular docking

The 3D structures of most EV71 viral proteins have been reported previously (Plevka et al., 2012). In this study, we used semi-flexible molecular docking program CDocker in Discovery Studio software package (Accelrys, San Diego, CA, United States) to evaluate the possible bindings between our compounds and EV71 viral proteins. CDocker was run with default parameters: random conformations and orientations were both set to “10”, the simulated annealing was set to “true”, “CHARMM forcefield” and “grid-based potential” were selected, the Top Hits was set to default value as “10”. After docking runs, the -CDocker Energy was interpreted as an approximated indicator of the binding affinity.

### Statistical analysis

*In vitro* experiment values are expressed as mean ± standard deviation (SD) from *n* = 3 biological repeats. Graphing and analysis were performed using GraphPad Prism v7. Statistical analysis was performed using one-way ANOVA. Statistical analysis for survival rate was performed using the Log-Rank (Mantel-Cox) test. Statistical analysis for clinical score was performed using Ridit assay with SigmaPlot 12.0. Statistical significance represented by asterisks is marked correspondingly in the figures, *p < 0.05, **p < 0.01, and ***p < 0.001. All experiments were independently repeated twice.

## Results

### Screening for antivirals against EV71 infection from botanical drug extracts

**FIGURE 1:**
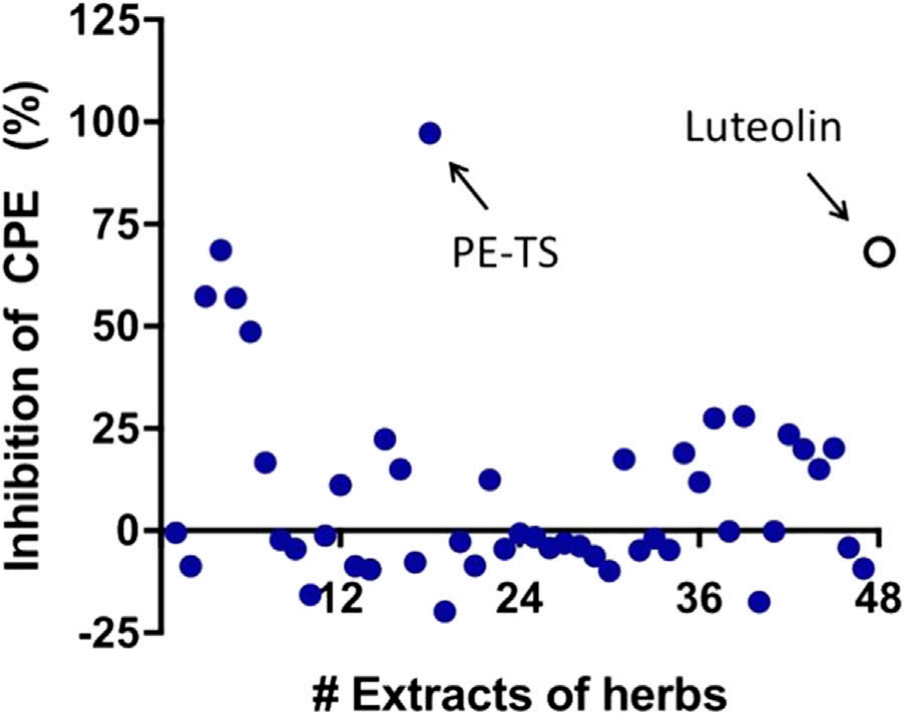
Screening for antivirals against EV71 infection from Mongolian botanical drug extracts. RD cells were infected with EV71 (MOI = 0.05) and treated with herbal extracts or luteolin as a positive control. The cell viability was measured at 48 hpi.

First, we screened the inhibitory effects of 47 botanical drug extracts on EV71 infection by measuring the CPE on RD cells. The cells were infected with EV71 (MOI = 0.05) and treated with botanical drug extracts or luteolin as a positive control, and were measured for cell viability at 48 hpi. The cell viability of the virus control group decreased to 31.45 ± 0.29% of the non-infection control group. Most botanical drug extracts did not show obvious antiviral effects (inhibition of CPE less than 30%), whereas four botanical drug extracts displayed antiviral effects against EV71 (inhibition of CPE greater than 50%) (Figure 1). Among them, the petroleum ether extract of Tournefortia sibirica L. (PE-TS) displayed the highest CPE inhibition against EV71 infection (97%) (Figure 1). As a positive control, the natural compound luteolin (8.59 μg/ml) showed 71% CPE inhibition. *T. sibirica*, which belongs to the family Boraginaceae, is utilized in Inner Mongolia of China as a remedy for scrofula, eczema, sores, and ulcers (Wu, 2014; Ao and Na, 2015). To date, no study has shown that *T. sibirica* extract has an inhibitory effect on enteroviruses. Therefore, PE-TS was the focus of this study as a novel potential antiviral agent against EV71 infection.

### PE-TS protected cells from EV71 or CVA16 infection

**FIGURE 2:**
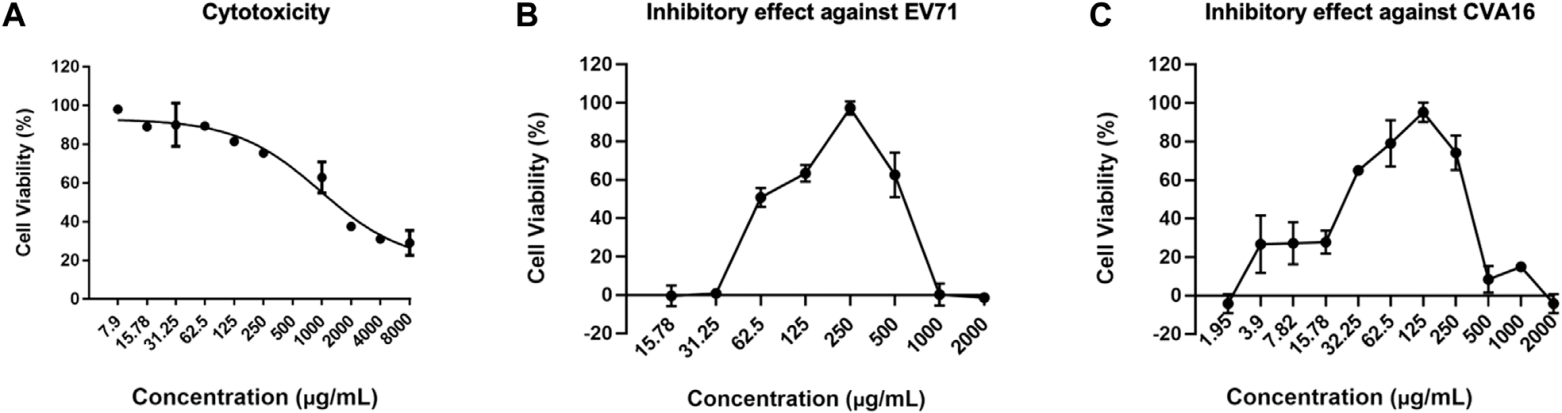
Cytotoxicity of PE-TS and inhibitory effect against EV71 or CVA16 infection in RD cells. **(A)** Cytotoxicity of PE-TS in RD cells was measured by cell viability assay after exposure for 48 h. The RD cells were infected with EV71 (MOI = 0.05) **(B)** and CVA16 (MOI = 0.005) **(C)** and exposed to PE-TS at the indicated concentrations. Inhibitory effect was measured by cell viability assay at 48 hpi. Data represent mean ± SD (*n* = 3).

Next, we determined the cytotoxicity of PE-TS in cells. RD cell viability decreased with increasing PE-TS concentration in a dose-dependent manner, with a CC_50_ of 970 ± 55.6 μg/ml (Figure 2A). The inhibitory effect of PE-TS on EV71- or CVA16-induced CPE in RD cells was also investigated. As shown in Figure 2B, the optimal protection rate of PE-TS against EV71 infection was 97.25 ± 1.47% at a concentration of 250 μg/ml. The EC_50_ against EV71 was 76.72 ± 8.03 μg/ml in RD cells. Selective index value was 12.6, suggesting the potent of development. The antiviral effect of PE-TS decreased at concentrations greater than 500 μg/ml, mainly due to its cytotoxicity. As shown in Figure 2C, the optimal protection rate of PE-TS against CVA16 infection was 94.75% ± 5.64% at a concentration of 125 μg/ml. The EC_50_ against CVA16 was 30.57 ± 4.26 μg/ml, and selective index value was 31.7.

### PE-TS inhibited EV71 genomic RNA replication and viral protein synthesis

**FIGURE 3:**
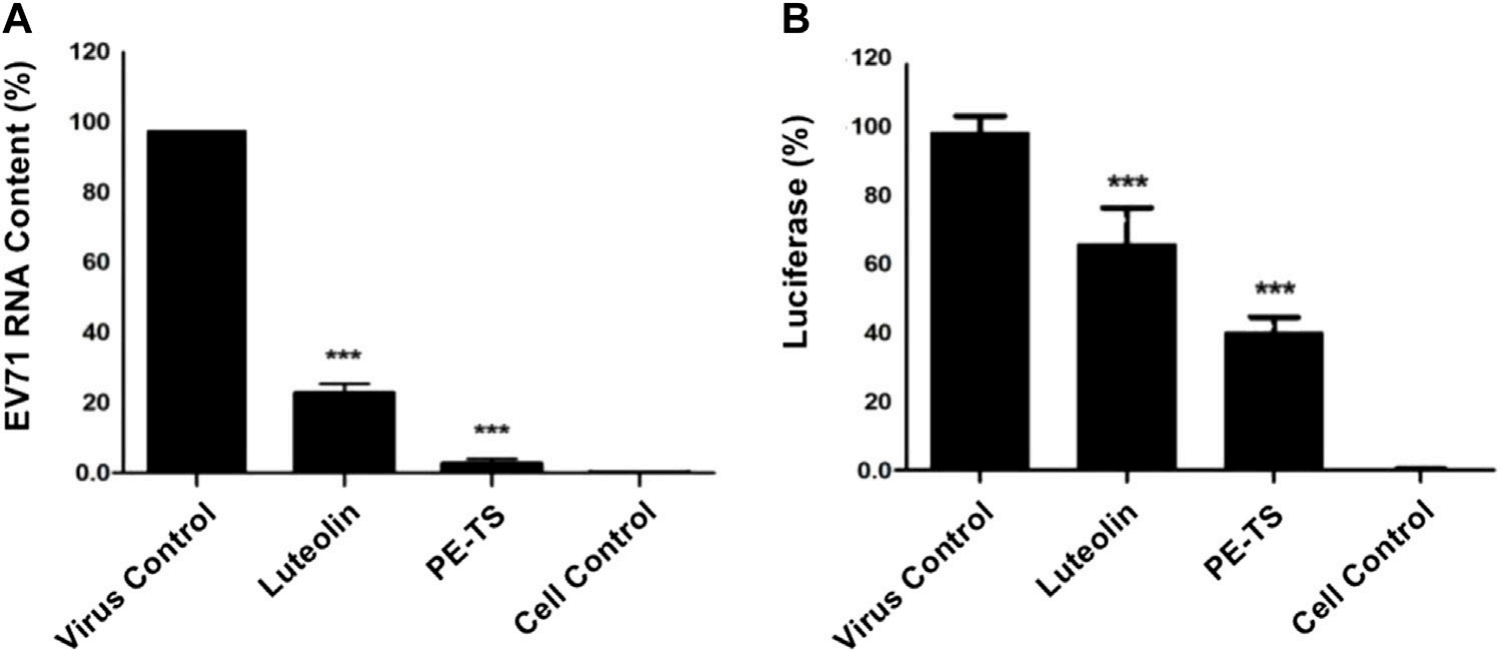
Inhibitory effects of PE-TS on EV71 genomic RNA replication and viral protein synthesis. **(A)** RD cells were infected with EV71 (MOI = 0.2) and treated with PE-TS (250 μg/ml). The supernatant was collected at 16 hpi for quantification of viral genomic RNA content using qRT-PCR. **(B)** RD cells were infected with EV71 reporter pseudovirus and treated with PE-TS (250 μg/ml). The intracellular luciferase activity was determined by luminescence reading at 16 hpi. Luteolin (8.59 μg/ml) was used as the positive control. Data represent mean ± SD (*n* = 3). Significance: ***p < 0.001 compared with virus control group.

The effects of PE-TS on the biosynthesis of EV71 were examined based on genomic RNA replication and viral protein synthesis at 16 hpi without an obvious cytopathic effect (Lu et al., 2011). PE-TS (250 μg/ml) had a significant inhibitory effect on EV71 RNA replication in RD cells, measured using qRT-PCR, with an inhibitory rate of 90.45% (Figure 3A). To evaluate viral protein synthesis in cells, we used an EV71 reporter pseudovirus carrying a luciferase coding sequence in the viral genome. Luciferase activity, which can be measured by luminescence, acts as an indicator of viral protein synthesis when the reporter pseudovirus infects cells. PE-TS showed a significant inhibitory effect on viral protein synthesis at a concentration of 250 μg/ml, with an inhibition rate of 62.54% (Figure 3B). As a positive control, luteolin treatment also significantly decreased viral RNA content and luciferase activity (Xu et al., 2014). These results indicate that the antiviral effects of PE-TS against EV71 were due to the inhibition of viral RNA replication and protein synthesis.

### PE-TS inhibited the generation of EV71 progeny virus

**FIGURE 4:**
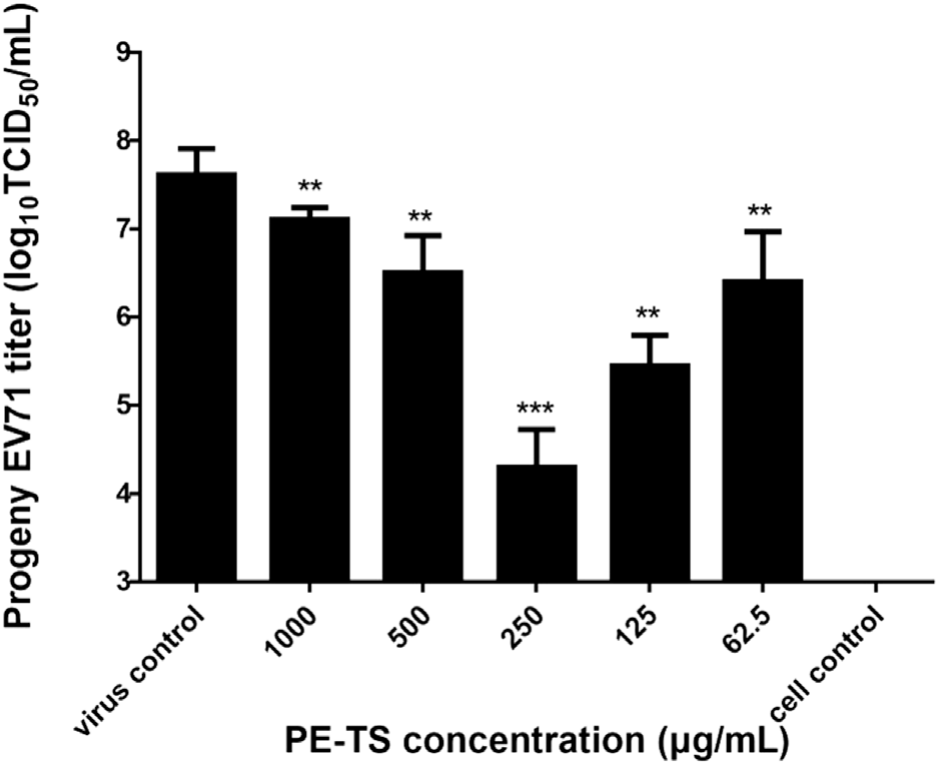
Inhibitory effects of PE-TS on the generation of EV71 progeny virus. RD cells were infected with EV71 (MOI = 0.2) and treated with PE-TS at the indicated concentrations (*n* = 3). The supernatant progeny virus titer (TCID_50_/ml) was measured at 16 hpi. Significance: **p < 0.01, ***p < 0.001 compared with virus control group.

To analyze the impact of PE-TS administration on the generation of EV71 progeny virus, the TCID_50_ titer of EV71 in the supernatant was detected at 16 hpi. The results showed that PE-TS decreased the extracellular EV71 titer by 3.75 log at a concentration of 250 μg/ml (Figure 4), which proved that PE-TS inhibited the generation of EV71 progeny viruses.

### PE-TS protected newborn mice from lethal EV71 challenge

**FIGURE 5:**
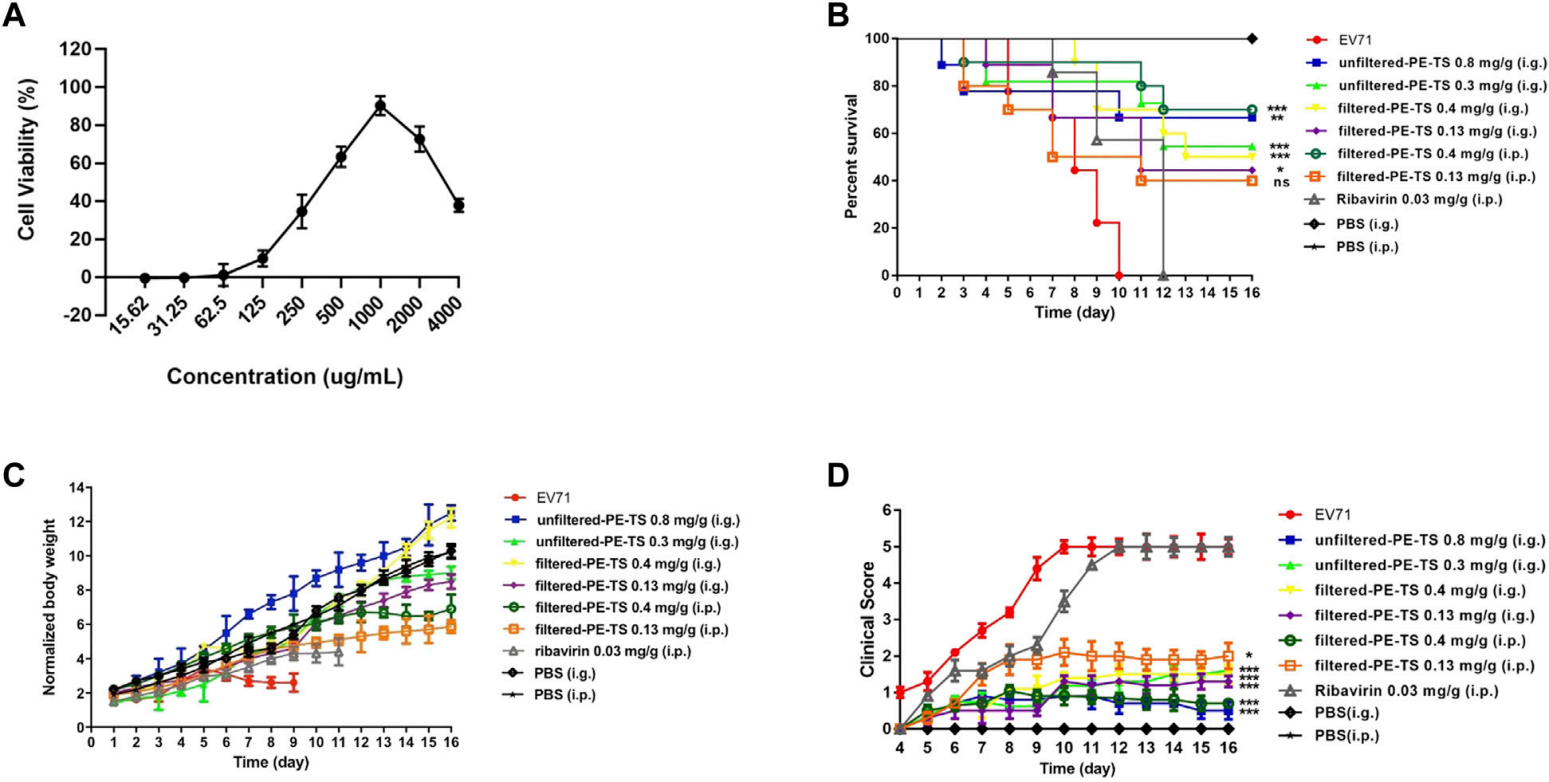
Protection by PE-TS treatment in newborn mice from lethal EV71 challenge. **(A)** Inhibitory effect of filtered-PE-TS against EV71 infection in RD cells. The RD cells were infected with EV71 (MOI = 0.05) and exposed to filtered-PE-TS at the indicated concentrations (*n* = 3). The inhibitory effect was measured by cell viability assay at 48 hpi. The experiments were repeated twice independently. BALB/c lactating mice (*n* = 9–11 per group) were intracranially challenged with EV71 at a dose of 1.5 × 10^6^ TCID_50_ within 24 h after birth and then administered PE-TS (unfiltered or filtered) *via* gavage or intraperitoneal injection at the indicated dose, respectively. Ribavirin (0.03 mg/g) was used as the medicine control group, and 10% DMSO was added with PBS as the non-infection control group. The mice were administered PE-TS once a day for seven consecutive days, and the survival rate **(B)**, body weight **(C)**, and clinical symptom scores **(D)** were recorded for 16 consecutive days post-EV71 challenge. Significance: *p < 0.1, **p < 0.05, ***p < 0.001 compared with virus control group.

We further investigated the protective effect of PE-TS against EV71 infection in a newborn mouse model (Dai et al., 2019). In this study, we aimed to simulate clinical oral administration and intravenous infusion by direct gavage or intraperitoneal injection, respectively. Considering the suspension character of PE-TS in a DMSO-water solution and its potential serious adverse outcome after peritoneal administration, PE-TS in the DMSO-water solution was filtered to remove insoluble matter. Subsequently, the *in vitro* antiviral activity and cytotoxicity of filtered PE-TS were determined. The results showed that the effective antiviral concentration of filtered PE-TS increased with an optimal antiviral concentration of 1,000 μg/ml (Figure 5A) compared with that of unfiltered PE-TS. The non-toxic concentration of filtered and unfiltered PE-TS were determined in 1-day-old BALB/c mice (Supplementary Figure S1).

One-day-old BALB/c mice were intracranially challenged with a lethal dose of EV71 and administered PE-TS treatment for seven days *via* gavage or intraperitoneal injection, respectively. The survival rate, clinical score, and body weight of mice were monitored daily for 16 days after the virus change. Several dose gradients for unfiltered PE-TS (i.g.) and filtered PE-TS (i.g. and i.p., respectively) treatments were set in this experiment. Ribavirin was used as a control because it is a broad-spectrum antiviral drug (Pang et al., 2014). As a result, intraperitoneal injection of 0.4 mg/g filtered-PE-TS and direct lavage of 0.8 mg/g unfiltered-PE-TS significantly improved the survival rate (70% and 66.7%, respectively; Figure 5B). They also significantly relieved clinical symptoms and decreased weight loss in the infected mice (Figures 5C,D). Consistent with previous studies (Dai et al., 2019; Kang et al., 2021), ribavirin treated mice exhibited only slight improvement of mortality. This is probably due to ribavirin may exhibit more cytotoxicity in the EV71-infected suckling mice model. These results demonstrate that PE-TS effectively protected newborn mice from lethal EV71 infection.

### Identification of chemical constituents of PE-TS by GC-MS

**FIGURE 6:**
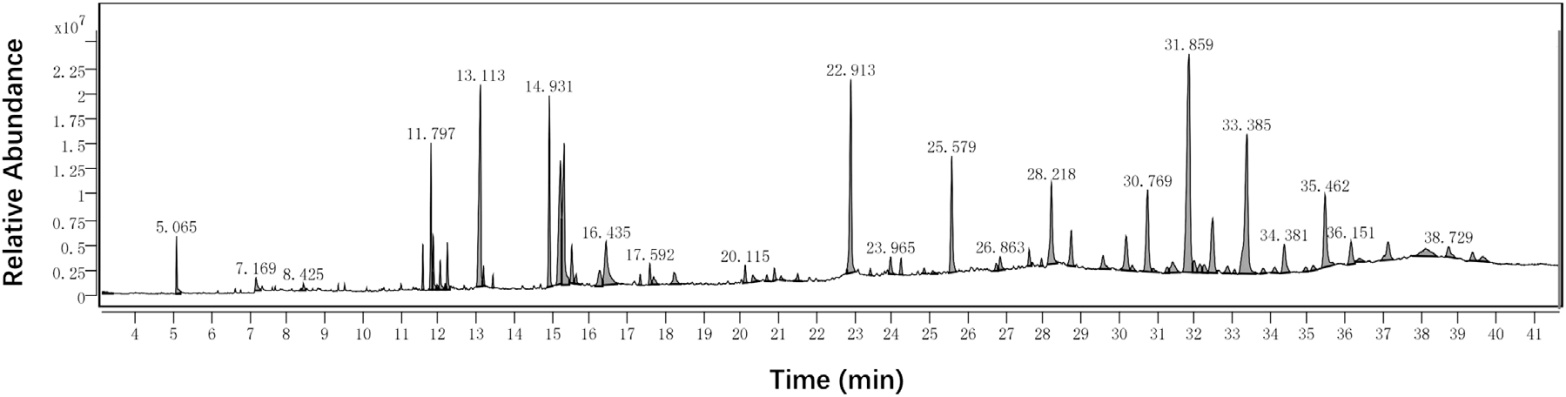
Total ion chromatogram of PE-TS analyzed by GC-MS.

The chemical constituents of PE-TS were further analysed by GC-MS of Single-Quadrupole systems. Such approach can preliminarily describe the profile of volatile organic compounds in PE-TS. The GC-MS total ion chromatograms of the chemical constituents of PE-TS were shown in Figure 6. The mass spectrum of each detected peak was retrieved in the reference standard spectral library NIST17.L. Sixty compounds were identified and analyzed from PE-TS (Supplementary Table S1), mainly including 15 esters, 13 hydrocarbons, 7 terpenoids, 5 ketones, 3 amides, 3 carboxylic acids, 2 nitrile compounds, and 12 compounds each belongs to unique type. None of these compounds has been reported anti-enterovirus activity from literature.

### Prediction of compound targets *via* network pharmacology

**FIGURE 7:**
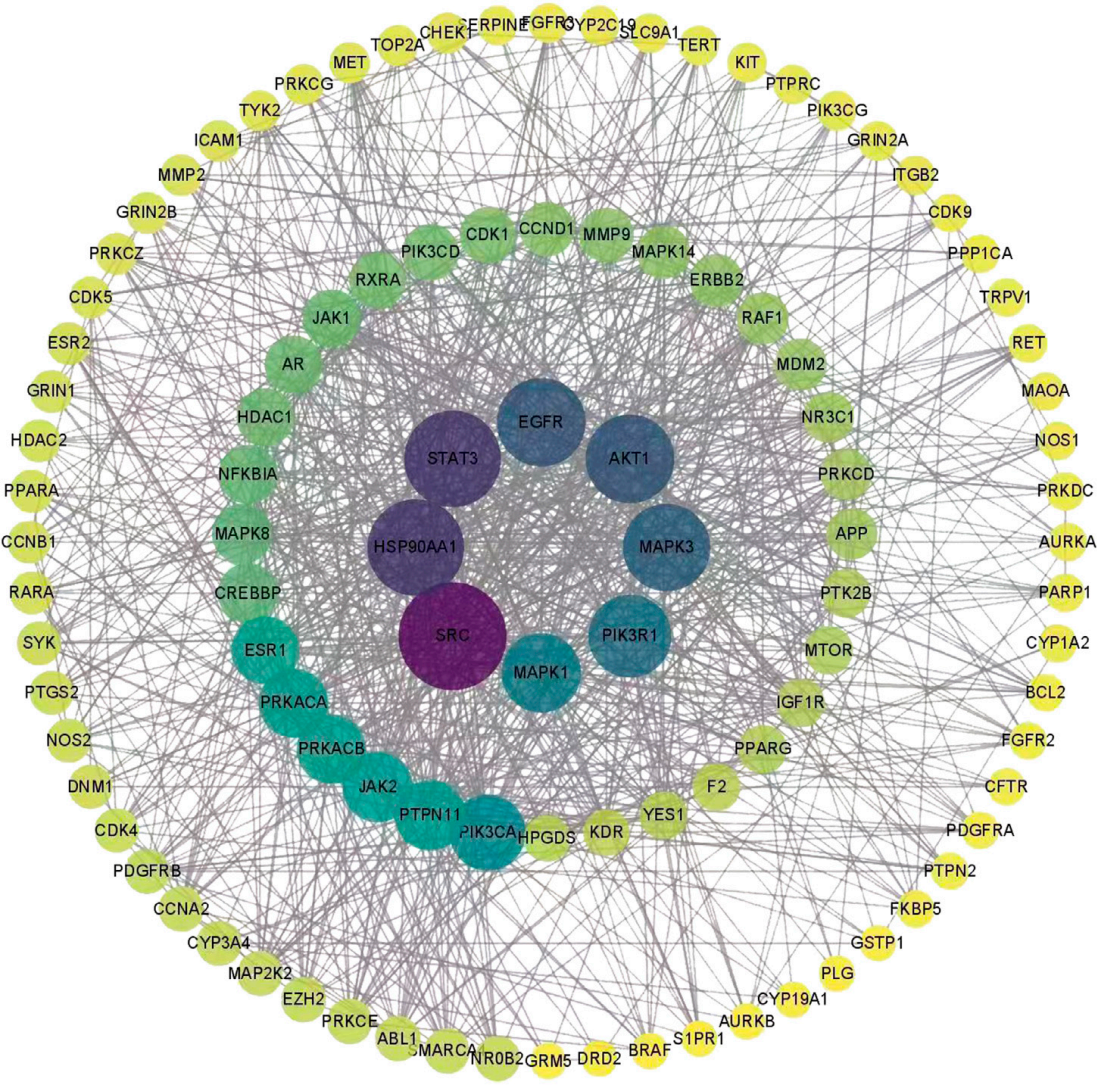
Compound-target network diagram of volatile organic compounds of PE-TS in the treatment of HFMD.

We firstly analyzed potential active compounds with oral bioavailability from volatile organic compounds of the PE-TS. Then, a total of 638 compound targets were identified from the potential active compounds. Further, a total of 5549 HFMD-related targets were acquired by screening the GeneCards and DisGeNET databases. After the intersection of 638 compound targets and 5549 HFMD-related targets, 346 overlapping targets were obtained. A protein-protein interaction network of 346 overlapping targets was established, containing 346 nodes and 1,694 edges. According to the twofold median degree value obtained by topological analysis, 8 core targets were further selected, including SRC, STAT3, HSP90AA1, AKT1, EGFR, MAPK3, PIK3R1, and MAPK1 (Figure 7).

### Docking simulation of volatile organic compounds of PE-TS with potential targets

**FIGURE 8:**
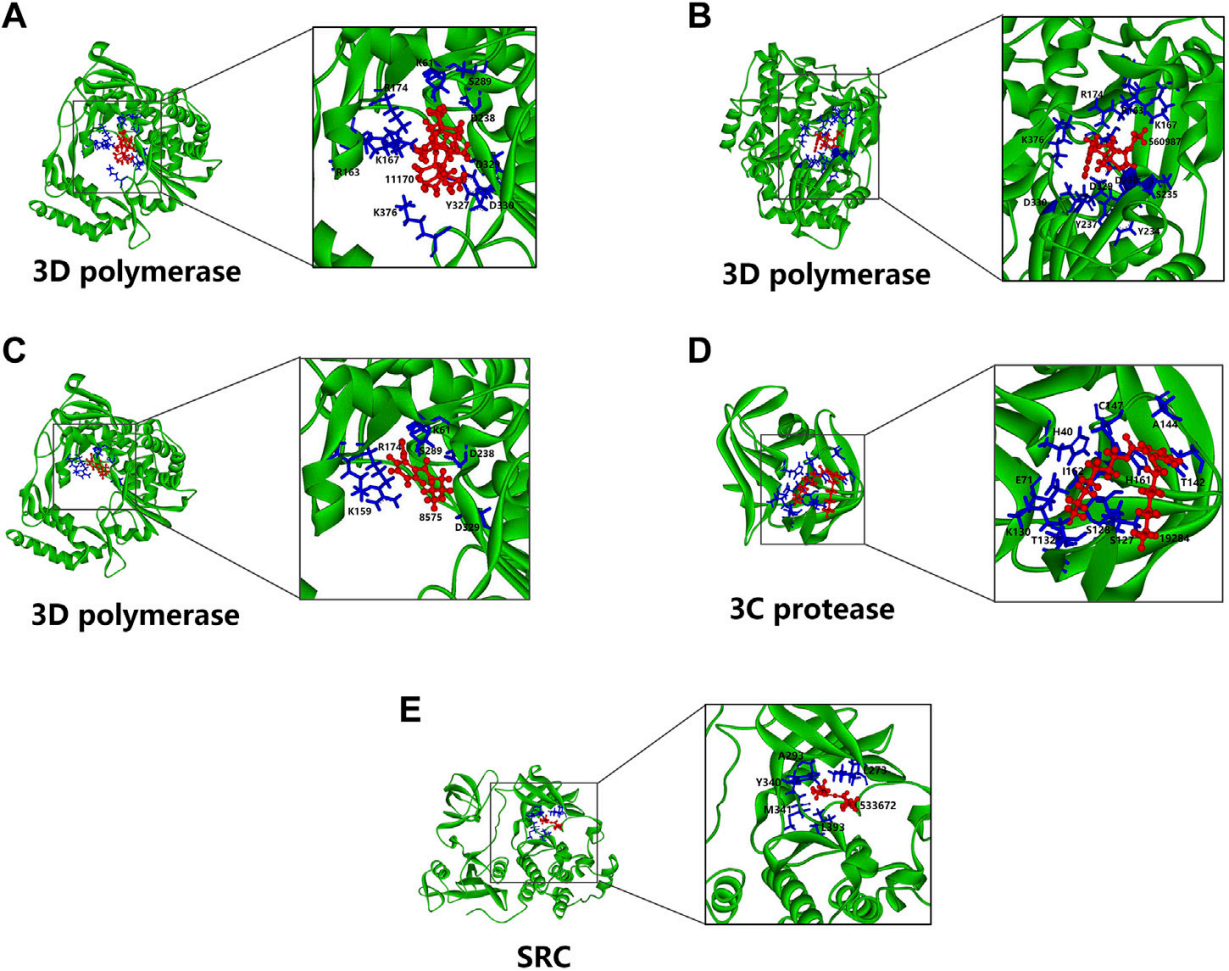
Molecular docking models of compounds with potential protein targets. **(A–C)** Hexadecamethyl cyclooctasiloxane, 3-thiazolidinecarboxylic acid and monobutyl phthalate bind to EV71 3D polymerase, respectively. **(D)** Diheptyl phthalate binds to EV71 3C protease. **(E)** Nona-2,3-dienoic acid, ethyl ester binds to SRC. The left side of all the panels show the whole models, the right side shows the zoomed-in display of the binding site. The proteins are shown in main chain ribbon mode (green), the compounds are shown in ball and sticks mode (red), and the amino acids residues participate in binding are shown in stick mode (blue) with labels.

To further predict the compound targets, molecular docking was performed. We focused on both EV71 proteins which are usually identified as inhibitor targets and host proteins which selected from compound-target interaction network analysis, as docking targets. Docking results showed potential targets, including EV71 3D polymerase, 3C protease and host protein SRC. EV71 3D protein is an RNA-dependent RNA polymerase which is responsible for viral RNA replication (McMinn, 2002). 3C protease is essential for cleavage of viral precursor polyproteins and also host proteins associated to antiviral responses (Li et al., 2002). SRC, namely steroid receptor coactivator, is a node of several signals involved in many biological processes such as proliferation, differentiation, motility and adhesion, and is also an important regulator of cell metabolism (Pelaz and Tabernero, 2022). As shown in Figure 8, hexadecamethyl cyclooctasiloxane (PubChem CID: 11170), 3-thiazolidinecarboxylic acid (PubChem CID: 560987), monobutyl phthalate (PubChem CID: 8575) efficiently binds to 3D polymerase with best -CDocker energy 74.283, 30.883 and 30.524 kcal/mol, respectively. Diheptyl phthalate (PubChem CID: 19284) efficiently binds to 3C protease with best -CDocker energy 41.453 kcal/mol. Furthermore, nona-2,3-dienoic acid, ethyl ester (PubChem CID: 533672) efficiently binds to SRC with best -CDocker energy 24.646 kcal/mol.

## Discussion

EV71 and CVA16 are the main pathogens causing HFMD. To date, no specific antiviral drug against enteroviruses has been approved for clinical use. Therefore, research and development regarding antiviral reagents for HFMD are urgently needed. The pharmacological activity of botanical drugs can be identified using modern scientific research methods to develop new drugs (Li and Weng, 2017).

In this study, we explored antiviral constituents from folk used botanical drugs among Mongolian community of China. The PE-TS identified from the screening presented promising antiviral activity against EV71 and CVA16 infection *in vitro*, with optimal inhibition rates of 97.25% (250 μg/ml) and 94.75% (125 μg/ml), respectively (Figure 2). The cytotoxicity of PE-TS was low, which is suitable for research and development of antiviral drugs. The preliminarily antiviral mechanisms of PE-TS were indicated as the inhibition of viral genome replication, protein synthesis (Figure 3), and the yield of progeny viruses of EV71 (Figure 4).

The protective effect of PE-TS against lethal dose EV71-challenged neonatal mice was further evaluated. Since traditional administration of botanical drugs in China is mainly oral decoction and the drug dosage is large, gavage administration may better simulate the pharmacokinetic process. The optimal protective rate of unfiltered-PE-TS by direct gavage in EV71-challenged newborn mice was 66.7% at a dose of 0.8 mg/g. We also established an intraperitoneal injection route of PE-TS treatment to simulate potential intravenous infusion in clinical administration, not only because of its direct absorption but also to provide the basis and pave the way for the development of injection. The filtered-PE-TS (0.4 mg/g) displayed a protective rate of 70% *via* intraperitoneal injection. These data suggest that PE-TS has the potential to be used as an antiviral botanical drug candidate against EV71 infection through either oral administration or intravenous infusion.

Multiple botanical drug extracts were identified the antiviral effects against EV71 infection *in vitro*, such as 95% ethanol extract of Paris polyphylla Smith (EC_50_ 78.46–125.00 μg/ml) (Wang et al., 2011), the water extract of Kalanchoe gracilis (EC_50_ 35.88 μg/ml) (Wang et al., 2012), Daphne Genkwa Sieb. et Zucc. (EC_50_ 163–824 μg/ml) (Chang et al., 2012), Salvia miltiorrhiza (EC_50_ 742 μg/ml for SA1, 585 μg/ml for SA2) (Wu et al., 2007), Houttuynia cordata Thunb. (EC_50_ 125.92 μg/ml) (Lin et al., 2009). Comparing with these botanical drug extracts, the effective concentration of PE-TS (EC_50_ 76.72 μg/ml) against EV71 *in vitro* is relatively low. Only a few studies evaluated antiviral activities of botanical drugs against EV71 in animal experiments. Chen et al. reported that the aqueous extract of Schizonepeta tenuifolia Briq (0.25 mg/g) protects mice (75% survival rate) from non-lethal EV71 challenge (40% survival rate of control group) (Chen et al., 2017). Zhang et al. reported that prescription Shouzuqing suppository (raw drug 11 0.05 mg/g) increases the survival of lethal EV71-challenged mice (protective rate approximately 50%) (Zhang et al., 2020). PE-TS showed comprehensive advantages in dose (0.8 mg/g by direct gavage or 0.4 mg/g *via* intraperitoneal injection) and protection rate from lethal challenge (66.7%–70%). In general, these studies all performed valuable explorations of the application of botanical drugs in anti-EV71 treatment.

There is no relevant literature about the chemical composition of PE-TS. We further analyzed PE-TS by GC-MS of Single-Quadrupole systems and identified 60 compounds (Supplementary Table S1), providing a preliminary overview of volatile components in PE-TS. The effective substances of PE-TS should be further studied by high resolution GC-MS, traditional column chromatography separation method, etc. Notably, some of the identified compounds from PE-TS were reported antiviral activities, such as the anti-HCoV-OC43 activity of α-amyrin (EC_50_ = 3.99 μM) (Zhao et al., 2022), the anti-CHIKV activity of β-Amyrone (EC_50_ = 86 μM) (Bourjot et al., 2012). Palmitic acid showed anti-HIV-1 and anti-HCV activity (Lee et al., 2009; Miyake et al., 2012). Molecular docking showed that β-sitosterol and bakuchiol interact with SARS-CoV-2 3CL protease and ACE2 receptor, respectively (Li et al., 2021b; Chowdhury, 2021). We further predicted potential targets of compounds derived from GC-MS by network pharmacology, and simulated potential binding of compounds with viral proteins and host proteins. The results implied that hexadecamethyl cyclooctasiloxane, 3-thiazolidinecarboxylic acid and monobutyl phthalate are inhibitors of EV71 3D polymerase; diheptyl phthalate is inhibitor of 3C protease; nona-2,3-dienoic acid, ethyl ester is inhibitor of host protein SRC, whose function defectiveness may impair proliferation and metabolism of host cell infected by EV71 so that decrease the generation of offspring virions.

Although PE-TS showed promising antiviral activities, the lack of exact underlying mechanisms is a limitation of this study. Botanical drugs are usually difficult to uncover molecular mechanisms of action because of complexity of constituents. The preliminary overview of volatile components in PE-TS, network pharmacology prediction and molecular docking are far not sufficient to describe the profile of antiviral mechanisms. The antiviral evaluation of five compounds predicted from network pharmacology prediction and molecular docking against EV71 infection are needed. More efforts should be paid to separate single compounds from PE-TS, and to determine which compounds are the antiviral drug substances of PE-TS, followed by uncovering their specific mechanisms in the life cycle of EV71.

The present study identified PE-TS for the first time as an effective antiviral botanical drug extract against EV71 infection *in vitro* and *in vivo* as well as against CVA16 infection *in vitro*, which provides a possible candidate for the development of HFMD antiviral drugs. In addition, this study encourages efforts that discover antiviral constituents from ethnic botanical drugs.

## Data Availability

The original contributions presented in the study are included in the article/Supplementary Material, further inquiries can be directed to the corresponding authors.
