# Supplementary material for: Antiviral effects of the petroleum ether extract of Tournefortia sibirica L. against enterovirus 71 infection in vitro and in vivo
Source: Front Pharmacol. 2022 Nov 29;13:999798. doi: 10.3389/fphar.2022.999798 (PMC9744809; doi:10.3389/fphar.2022.999798)
Supplement: Supplementary file 1 [file Table1.DOC]

Supplemental Table 1. Volatile organic compounds of the PE-TS detected by GC-MS

| NO. | Retention time (min) | Formula | MW | Compound | Peak area (%) | PubChem (CID) |
| --- | --- | --- | --- | --- | --- | --- |
| 1 | 3.120 | C_8_H_16_ | 112.21 | Ethylcyclohexane | 1.16 | 15504 |
| 2 | 3.589 | C_8_H_17_N_3_ | 155.241 | imino-octylimino-azanium | 0.15 | 6332335 |
| 3 | 4.360 | C_4_H_10_O_4_ | 122.12 | 1,2,3,4-Butanetetrol [s-R* R*]- | 0.51 | [8998](https://pubchem.ncbi.nlm.nih.gov/compound/8998) |
| 4 | 4.734 | C_6_H_11_N_3_ | 125.172 | Azidocyclohexane | 0.16 | 88153 |
| 5 | 5.318 | C_9_H_8_ | 116.16 | 1-Phenylallene | 0.16 | 75355 |
| 6 | 5.693 | C_8_H_7_N | 117.148 | O-Tolyl Isocyanide | 0.87 | 575991 |
| 7 | 5.754 | C_8_H_13_NO_2_ | 155.19 | 2,7-Dioxatricyclo[4.4.0.0<3.8>]decan-4-amine,stereoisomer | 0.15 | 551612 |
| 8 | 6.304 | C_6_H_8_O_4_ | 144.1253 | 4H-Pyran-4-one,2,3-dihydro-3,5-dihydroxy-6-methyl- | 0.23 | 119838 |
| 9 | 6.806 | C_10_H_8_ | 128.171 | Naphthalene | 1.03 | 931 |
| 10 | 7.268 | C_17_H_23_NO_5_S | 353.4 | [3-Thiazolidinecarboxylic Acid](https://pubchem.ncbi.nlm.nih.gov/compound/560987) | 0.29 | 560987 |
| 11 | 7.985 | C_10_H_14_N_20_ | 178.231 | 2-diazonio-4,7,7-trimethylbicyclo[2.2.1]hept-2-en-3-olate | 0.28 | 572114 |
| 12 | 8.673 | C_6_H_14_BNO | 126.993 | 2-Butyltetrahydro-1,3,2-oxazaborole | 0.27 | 558978 |
| 13 | 8.767 | C_15_H_14_N_4_O_3_ | 298.297 | 3-amino-8-hydroxy-9-methyl-1,6-dioxo-8-phenyl-2,7-diazaspiro[4.4]non-3-ene-4-carbonitrile | 0.40 | - |
| 14 | 8.949 | C_15_H_26_O | 222.37 | β-Acorenol | 0.99 | 6430766 |
| 15 | 9.081 | C_6_H_5_BO_3_ | 135.913 | 2-hydroxy-1,3,2-benzodioxaborole | 0.20 | 585956 |
| 16 | 9.422 | C_14_H_12_Br_2_N_2_O_2_ | 400.065 | 2,3-dibromo-N-(naphthalen-1-ylcarbamoyl)propanamide | 0.60 | 21423253 |
| 17 | 10.001 | [C](https://pubchem.ncbi.nlm.nih.gov/#query=C14H21BO3)_14_H_21_BO_3_ | 248.13 | 2-Butyne, 4-Acetoxy-1-(9-Borabicyclo[3.3.1]Non-9-Yl)Oxy- | 0.60 | [538992](https://pubchem.ncbi.nlm.nih.gov/compound/538992) |
| 18 | 10.172 | C_9_H_18_FO_2_P | 208.21 | Phosphonofluoridic Acid, (1-Methylethyl)-, Cyclohexyl Ester | 0.16 | 581236 |
| 19 | 10.629 | C_12_H_20_OSi | 208.37 | Triisobuty(3-phenylpropoxy)silane | 0.19 | - |
| 20 | 10.866 | C_16_H_48_O_8_Si_8_ | 593.232 | Cyclooctasiloxane,hexadecamethyl- | 0.20 | 11170 |
| 21 | 11.191 | C_8_H_16_N_2_O_7_ | 252.222 | cycasin | 0.19 | 5459896 |
| 22 | 11.251 | C_21_H_38_O_2_ | 322.5252 | [1,1´-Bicyclopropyl]-2-octanoic acid,2´-hexyl-,methyl ester | 0.44 | 552098 |
| 23 | 11.576 | C_10_H_15_PS | 198.265 | ethyl ester of ethylphenylthiophosphinic acid | 0.38 | 12590706 |
| 24 | 11.824 | [C_11_H_18_N_2_O_2_](https://pubchem.ncbi.nlm.nih.gov/) | 210.27 | Acetamide,N-methyl-n-[4-(3-hydroxypyrrolidinyl)-2-butynyl]- | 0.16 | 536669 |
| 25 | 12.122 | C_11_H_16_O_3_ | 196.24 | 6-Hydroxy-4,4,7a-trimethyl-5,6,7,7a-tetrahydrobenzofuran-2(4H)-one | 0.91 | 14334 |
| 26 | 12.309 | C_12_H_16_O_5_ | 240.25 | 2-(2-Carboxyethyl)-6,6-dimethyl-3-oxocyclohex-1-enecarboxylic acid | 0.17 | 534589 |
| 27 | 12.739 | C_20_H_38_ | 278.516 | Neophytadiene | 4.95 | 10446 |
| 28 | 12.827 | C_16_H_31_BO | 250.228 | Diethyl(1-cyclododecen-1-yloxy)borane | 1.98 | 5367718 |
| 29 | 13.075 | C_15_H_27_BO_2_ | 250.19 | 9-Borabicyclo[3.3.1]nonane,9-(3-methoxycyclohexyl)oxy- | 0.78 | 558071 |
| 30 | 13.339 | C_10_H_16_O | 152.233 | 2,2-Dimethylocta-3,4-Dienal | 1.47 | 253228 |
| 31 | 13.989 | C_16_H_25_NO_3_S | 311.4 | 1-(10,10-Dimethyl-3,3-dioxo-3-thia-4-azatricyclo[5.2.1.0<1,5>]dec-4- yl)-3-methylpent-4-en-1-one | 0.21 | 550228 |
| 32 | 14.044 | C_12_H_14_O_4_ | 222.237 | Monobutyl phthalate | 0.18 | 8575 |
| 33 | 14.601 | C_16_H_32_O_2_ | 256.424 | Palmitic acid | 14.35 | 985 |
| 34 | 14.799 | C_22_H_34_O_4_ | 362.503 | Diheptyl phthalate | 1.37 | 19284 |
| 35 | 15.289 | C_18_H_36_O_2_ | 284.4772 | Ethyl hexadecanoate | 0.42 | 12366 |
| 36 | 16.633 | C_18_H_24_O | 256.3826 | Bakuchiol | 0.21 | [**5468522**](https://pubchem.ncbi.nlm.nih.gov/compound/5468522) |
| 37 | 17.807 | C_20_H_40_O | 296.531 | Phytol | 7.69 | 5280435 |
| 38 | 18.154 | C_19_H_34_O_2_ | 294.4721 | Sterculic acid | 2.95 | 12921 |
| 39 | 18.302 | C_12_H_20_ | 164.287 | cyclododecyne | 10.95 | 136909 |
| 40 | 18.710 | C_11_H_18_O_2_ | 182.26 | Nona-2,3-dienoic acid,ethyl ester | 1.63 | 533672 |
| 41 | 18.914 | C_15_H_26_O_2_ | 238.37 | Methyl 12,13-tetradecadienoate | 0.14 | 91692385 |
| 42 | 19.531 | C_10_H_19_O_12_PS | 273.2 | Phosphine sulfide,dichloromenthyl- | 0.92 | 535304 |
| 43 | 19.707 | C_12_H_22_O | 182.302 | ethyl linalyl ether | 0.51 | 175211 |
| 44 | 19.812 | C_15_H_25_NO_6_ | 315.362 | Indicine N-oxide | 1.55 | 280564 |
| 45 | 21.156 | C_27_H_40_O_4_ | 428.61 | Spirost-8-en-11-one,3-hydroxy-,<3β,5α,14β,20β,22β,25R>- | 0.50 | 22296114 |
| 46 | 21.426 | C_14_H_16_O_2_S_2_ | 280.4 | 4-(2-Phenyl-1,3-dithian-2-yl)oxolan-2-one | 0.26 | 323449 |
| 47 | 21.751 | C_13_H_25_BO | 208.148 | 9-Borabicyclo[3.3.1]nonane, 9-(2-methylbutoxy)- | 0.41 | 551243 |
| 48 | 22.258 | C_11_H_18_O_2_ | 182.26 | Nona-2,3-dienoic acid,ethyl ester | 0.16 | 533672 |
| 49 | 22.461 | C_12_H_22_O | 182.302 | 5,9-dimethyl-4,8-decadien-3-ol | 0.27 | 5365900 |
| 50 | 22.599 | C_10_H_14_N_2_O_2_ | 194.23 | 2-Cyclopropyl-4-(dimethoxymethyl)pyrimidine | 0.28 | 17750124 |
| 51 | 23.477 | C_15_H_26_ | 206.367 | Bicyclo[2.2.1]hept-2-ene, 5-octyl- | 0.25 | 15701270 |
| 52 | 23.789 | C_27_H_44_O_3_ | 416.636 | Calcitriol | 0.15 | 5280453 |
| 53 | 24.345 | C_27_H_40_O_4_ | 428.61 | Hydroxyprogesterone caproate | 0.27 | 169870 |
| 54 | 24.637 | C_26_H_44_O_5_ | 436.625 | Ethyl iso-allocholate | 0.17 | 6452096 |
| 55 | 24.962 | C_14_H_24_O_2_ | 224.34 | 2-(4-methylcyclohex-3-en-1-yl)propan-2-yl butanoate | 0.25 | 578423 |
| 56 | 28.13 | C_48_H_99_BO_3_ | 735.109 | Trihexadecyl borate | 6.70 | 292409 |
| 57 | 29.915 | C_29_H_50_O | 414.707 | β-Sitosterol | 9.11 | 222284 |
| 58 | 30.278 | C_30_H_48_O | 424.702 | β-Amyrone | 1.59 | 6454747 |
| 59 | 31.005 | C_39_H_76_O_3_ | 593.019 | 1,2-Dioleyl-sn-glycerol | 2.12 | 54606114 |
| 60 | 31.821 | C_30_H_50_O | 426.72 | α-Amyrin | 11.02 | 73170 |

“-”: No PubChem (CID) record.
