# Supplementary material for: Antiviral effects of the petroleum ether extract of Tournefortia sibirica L. against enterovirus 71 infection in vitro and in vivo
Source: Front Pharmacol. 2022 Nov 29;13:999798. doi: 10.3389/fphar.2022.999798 (PMC9744809; doi:10.3389/fphar.2022.999798)
Supplement: Supplementary file 2 [file Image1.pdf]

Supplemental Figure 1

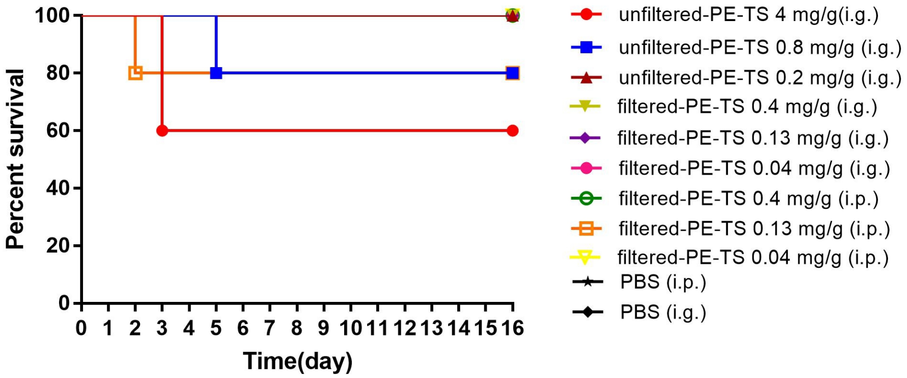

Supplemental Figure 1. Survival rate of suckling mice administrated with PE-TS. Newborn BALB/c mice (born within 24 h) (n = 5 per group) were intraperitoneal injection or direct lavage of indicated concentration of filtered or unfiltered PE-TS. All extracts were added to sterile PBS supplemented with 10% DMSO, which was administrated once daily for seven consecutive days. Two groups were inoculated with sterile PBS supplemented with 10% DMSO as negative controls. The mice were monitored daily for 16 days to observe survival rate.
